# Combined effects of education level and perceived social class on self-rated health and life satisfaction: Results of Korean labor and income panel study wave 8-wave 15

**DOI:** 10.1186/s12955-015-0375-5

**Published:** 2015-11-02

**Authors:** Jae-Hyun Kim, Ki-Bong Yoo, Eun-Cheol Park, Sang Gyu Lee, Tae Hyun Kim

**Affiliations:** Department of Public Health, Graduate School, Yonsei University, Seoul, Republic of Korea; Institute of Health Services Research, Yonsei University, Seoul, Republic of Korea; Department of Healthcare Management, Eulji University, Sungnam, Republic of Korea; Department of Preventive Medicine, Yonsei University College of Medicine, Seoul, Republic of Korea; Department of Hospital management, Graduate School of Public Health, Yonsei University, Seoul, Republic of Korea

**Keywords:** Education, Perceived social class, Self-rated health, Life satisfaction

## Abstract

**Background:**

To examine the combined effects of education level and perceived social class on self-rated health and life satisfaction in South Korea.

**Methods:**

We used data drawn from the 8 to 15th wave of the Korean Labor and Income Panel Study (KLIPS). Using wave 8 at baseline, data included 11,175 individuals. We performed a longitudinal analysis at baseline estimating the prevalence of self-rated health and life satisfaction among individuals by education level (high, middle, and low education level) and perceived social class (high, middle, and low social class).

**Results:**

For self-rated health, odds ratio (OR) of individuals with low education and low perceived social class was 0.604 times lower (95 % CI: 0.555–0.656) and the OR of individuals with low education and middle perceived social class was 0.853 time lower (95 % CI: 0.790–0.922) when compared to individuals with high education and high perceived social class. For life satisfaction, OR of individuals with low education and low perceived social class was 0.068 times lower (95 % CI: 0.063–0.074) and the OR of individuals with middle education and middle perceived social class was 0.235 time lower (95 % CI: 0.221–0.251) compared to individuals with high education and high perceived social class.

**Conclusions:**

This study shows that the combined effects of education level and perceived social class associated with self-rated health and life satisfaction. Our study suggests increasing education level and perceived social class. Additionally, it will be important to develop multi-dimensional measurement tools including education level and subjective social class.

**Electronic supplementary material:**

The online version of this article (doi:10.1186/s12955-015-0375-5) contains supplementary material, which is available to authorized users.

## Background

A number of social scientists have observed that a positive relationship exists between socioeconomic status (SES) indicators and health outcomes [1, 2]. Social, demographic, economic, and behavioural risk factors play an integral part in shaping one’s health and life satisfaction. These factors known as ‘social determinants of health’ do not exist in isolation from one another, but combine to determine the health of individuals, communities, and populations [3, 4]. House [5] presents a theoretical model linking social determinants with health. Social structural factors, such as sociodemographic and socioeconomic status, are considered underlying determinants of health. These factors are hypothesised to influence health primarily through their effects on proximate determinants such as psychosocial and behavioural risk factors. Accordingly, sociodemographic factors (e.g. sex, age, race, and nativity) interact with socioeconomic factors and influence one’s exposure to social stressors, health practices and behaviours, access to medical care and insurance, and ultimately health. However, more recently investigators have focused on perceived social class as a predictor of health. Multiple studies demonstrate that perceived social class continues to predict health once traditional measures of SES, like income and education, have been taken into account [6, 7].

One study showed that perceived social class was more consistently and strongly related to health-related factors than traditional measures of SES, such as income and education [8]. Another study showed that perceived social class can predict change in health status [7]. However, there is little agreement on which indicators should be used for examining the association between SES and health outcome. As a result, recent studies [9, 10] suggest the use of a combined effect of SES on health outcome. In addition, although the SES gradient in health has been studied widely, how social class causes poorer health is not clear. This may be, in part, because social class has been defined as SES, which is an external, purely objective measure that does not account for subjective, internalised perceived social class.

One previous study [11] suggested that these perceptions of relative ranking may be more important determinants of health than traditional measures, such as level of education and income, which assess material resources [12]. However, many previous studies used both objective and perceived social class indicators. In addition, despite the vast literature on the effect of SES on health, little is known about people’s perceptions of their placement in the social hierarchy, what determines these perceptions, or how these perceptions relate to health.

Although routine analysis using conceptually coherent and consistent measures of social class remains rare, income, deprivation, wealth, and education have typically been used to measure social class [9]. There have been many studies showing positive associations between traditional measures (i.e. education) and health outcomes, and between perceived social class and health outcomes.

Because education is easy to measure and applicable to persons, level of education is among the most widely used indicators of socioeconomic status in US public health research. Education level among adults who have completed their schooling, for example, is not affected by occurrence of serious illness, which can force individuals to work at jobs below the level of their normal occupations or otherwise cause their income to decline. Selection of education as a practical measure of socioeconomic position for the 1989 revision of the US standard death certificate was based on these considerations [13].

Especially, education level as traditional measures is a strong and consistent predictor of health outcomes [14]. Self-rated health has been found to be a good predictor of morbidity and mortality, and traditional measures of SES (e.g. education) are associated with self-rated health [15]. Those factors are related to life satisfaction. However, no study to date has examined the combined effect of education and perceived social class on self-rated health and life satisfaction. Therefore, in this study we investigated the size of combined effects of education level and perceived social class on self-rated health and life satisfaction in South Korea.

## Methods

Data were drawn from the 8 to 15th wave of the Korean Labor and Income Panel Study (KLIPS) that included various social classes. The survey passed an ethical review process by Statistics Korea, a central government organization for statistics. This study did not require an ethical review as the KLIPS dataset was publicly open and lacked information for individual identification.

The KLIPS is a longitudinal study of a representative sample of Korean households and individuals living in urban areas. The sampling method used at baseline was a two-stage stratified cluster sampling [16]. Households were selected after selecting the region. However, when considering the relatively low population ratio in rural regions of Korea (i.e. gun, eup, and myeon areas: the population ratio of gun, eup, and myeon areas was analysed as 18.0 % in the 2010 Population Census), it can be said that this study holds significant value as representative data for Korea. To give an equal probability of being sampled, weights were assigned to each respondent, enabling the results to represent the entire Korean population. This weighting method guarantees unbiased point estimates of population parameters for the entire population and its subsets.

As a type of study that possesses both the strengths of cross-sectional data and time series data, the KLIPS was constructed by repeatedly surveying the identical content for the same respondents every year. Thus, all variables surveyed by the KLIPS were repeatedly measured from the 1st wave to the 15th wave to collect observation cases at multiple points in time. Five thousand households in an urban region were selected, excluding households within a rural region. All household members residing in the relevant households form the panel sample, with repeated observations on the selected sample once a year every year.

Since 1998, the KLIPS has involved longitudinal research with annual assessments, tracing factors associated with labour movement and economic activities as well as income, expenditures, education, job training, and social activities of individuals. The original sample of the KLIPS (5,000 households) were sampled by two-stage stratified clustering, which first involved selection of the enumeration districts then selection of the households. Following cases can be added to KLIPS after survey started: 1) people in selected households who became 15 years-old, 2) people who were targeted for the survey but had not responded and became able to complete the survey, and 3) people who were over 15 years old that were newly added from selected households.

We only included participants from the 8 to 15th wave of KLIPS that had all the information needed for the study. Among the Wave 8 data consisting of 11,580 individuals, we excluded 405 individuals without information. Thus, the Wave 8 data included 11,175 individuals. The remaining Waves and number of individuals included are as follows: 11,487 of 11,756 in Wave 9, 11,487 of 11,855 in Wave 10, 11,606 of 11,734 in Wave 11, 14,355 of 14,489 in Wave 12, 14,094 of 14,118 in Wave 13, 13,778 of 13,900 in Wave 14, and 13,866 of 14,000 in Wave 15.

## Study variables

### Dependent variables

Self-rated health was measured by the question: ‘How do you rate your health compared with your age peers?’ The item was rated 1 (Very bad) to 5 (Very good). The reliability of the self-rated health question is found to be as good as or even better than that of most of the more specific questions on health [17]. Moreover, the question is closely related to health outcomes [17].

Life satisfaction was measured by the question: ‘How satisfied are you at present with your life?’ The item was rated 1 (very bad) to 5 (very good). Single-item measures of life satisfaction have previously been effectively used [18]. For both dependent variables, the response of ‘very bad’ or ‘bad’ indicated ‘bad’, and the response of ‘normal’ indicated ‘normal’, the response of ‘good’, or ‘very good’ indicated ‘good’.

### Independent variables

#### Education level

Education level was categorised into three groups: middle school or lower (Low), high school (Middle), and college or higher (High).

#### Perceived social class

Perceived social class was measured by asking the respondents to assess their social class based on the following question: ‘Where do you think you belong upon consideration of income, job, education level, and assets’. The item was rated 1 (Very Low) to 6 (Very High). The response of ‘very low’ or ‘low’ indicated ‘Low’. The response of ‘middle-low’ or ‘middle-high’ indicated ‘Middle’. The response of ‘high’ or ‘very high’ indicated ‘High’.

## Combined effects of education level and perceived social class

Combined effects represent the difference between the education level and perceived social class. We categorised the nine groups as follows: 1) high education and low perceived social class, 2) high education and middle perceived social class, 3) high education and high perceived social class, 4) middle education and low perceived social class, 5) middle education and middle perceived social class, 6) middle education and high perceived social class, 7) low education and low perceived social class, 8) low education and middle perceived social class, and 9) low education and high perceived social class. Thus, we analysed combined effects of education level and perceived social class on self-rated health and life satisfaction.

## Control variables

Age was divided into six categories: ≤29, 30–39, 40–49, 50–59, 60–69, and ≥70 years. Residential regions were categorised as Capital (Seoul), big city (Daejeon, Daegu, Busan, Incheon, Kwangju, or Ulsan) or small city (areas not classified as a big city). Individuals were classified as currently married or never married, with the latter group including those previously married, widowed, or divorced. Shift work was divided into two categories: yes or no (including housewives and students).

We included two daily life restriction variables: sensory system inability and physical inability. Activity restriction 1 indicated difficulties in learning, memory, and concentration; activity restriction 2 indicated difficulties in dressing and taking a bath; activity restriction 3 indicated difficulties in shopping and going to hospital; and activity restriction 4 indicated difficulties in occupation activity. Responses to these questions were categorised as ‘yes’ or ‘no’ in the questionnaire.

## Analytical approach and statistics

The Chi-square test and a longitudinal data analysis were used to investigate the impact of the gap between SES and perceived social class on self-rated health and life satisfaction. We ran Generalised estimation equation(GEE). Because our dependent variables were divided in three ordinal categories, *Proc Genmod with cumulative logit* in the SAS was used. The strength of GEE is that it is a very flexible approach to analyse correlated data from the same subjects over time [19, 20]. It controls for characteristics that change over time, such as confounding variables other than gender. For all analyses, the criterion for significance was a two-tailed *p*-value ≤ 0.05. All analyses were conducted using the SAS statistical software package version 9.4(SAS Institute Inc., Cary, NC, USA).

Self-rated health and life satisfaction (good/normal/bad) were the outcome variables in all GEE models. Covariates of interest from all subjects were added to the model to determine their effects on the probability of reporting self-rated health and life satisfaction. To determine whether the probability of self-rated health and life satisfaction changed over time, we included time (year) in the model as a categorical covariate; the regression coefficient was used to estimate both the change in probability of self-rated health and life satisfaction and independent variables, annually [21].

## Results

Table 1 lists the general characteristics of the variables of interest at baseline (Wave 8) for the 11,175 participants included in our analysis. Table 2 shows the general characteristics of the covariates according to the presence of self-rated health and life satisfaction class at baseline (Wave 8). The baseline weighted prevalence of self-rated health and life satisfaction class per year among individuals whose education matched their perceived social class was 12.9 % (high education and high social class), 14.0 % (medium education and medium social class), and 16.0 % (low education and low social class).

**Table 1:** General characteristics of interesting variables at baseline (Wave 8)

	Total	Weighted %
Combined effects of education level and subjective social class		
LL	1,994	16.0
LM	1,621	13.9
LH	807	8.8
ML	1,130	8.7
MM	1,674	14.0
MH	1,633	16.3
HL	395	3.2
HM	685	6.3
HH	1,236	12.9
Subjective social class		
Low	4,422	27.9
Middle	4,437	34.1
High	2,316	38.0
Education		
≤Middle school	3,519	38.6
High school	3,980	38.9
≥College	3,676	22.4
Age		
≤29	2,730	27.9
30–39	2,477	20.5
40–49	2,216	20.7
50–59	1,659	13.8
60–69	1,198	9.8
≥70	895	7.4
Gender		
Male	5,364	50.9
Female	5,811	49.1
Residential region		
Capital	2,561	25.0
Big city	3,322	29.0
Small city	5,292	45.9
Marital status		
Single	4,129	42.0
Married	7,046	58.0
Shift work		
Yes	412	3.6
No	3569	32.4
Not employed	7194	64.0
Smoking status		
Smoker	2,708	24.6
Former smoker	1,587	14.7
Never	6,880	60.8
Alcohol use		
Yes	6,260	56.6
Former user	1,210	10.3
No	3,705	33.1
Sensory system inability		
Yes	280	2.6
No	10,895	97.4
Physical inability		
Yes	945	8.1
No	10,230	91.9
Activity restriction 1		
Yes	535	4.8
No	10,640	95.2
Activity restriction 2		
Yes	266	2.5
No	10,909	97.5
Activity restriction 3		
Yes	487	4.3
No	10,688	95.7
Activity restriction 4		
Yes	1,136	9.8
No	10,039	90.2
Self-rated health		
Good	9,386	84.7
Bad	1,789	15.3
Life satisfaction		
Good	10,182	91.0
Bad	993	9.0
Total	11,175	100.0

**Table 2:** General characteristics of interesting variables at baseline (Wave 8) according to self-rated health and life satisfaction

	Self-Rated Health					Life Satisfaction				
	Good		Bad			Good		Bad		
	*N*	Weighted	*N*	Weighted	*P*-value	*N*	Weighted	*N*	Weighted	*P*-value
		%		%			%		%	
Combined effects of education level and subjective social class					<.0001					<.0001
LL	1,126	54.6	868	45.4		1,553	77.0	441	23.0	
LM	1,397	86.2	224	13.8		1,320	80.5	301	19.5	
LH	755	92.6	52	7.4		696	85.5	111	14.6	
ML	838	73.8	292	26.2		1,094	97.1	36	3.0	
MM	1,552	92.2	122	7.9		1,620	96.9	54	3.1	
MH	1,577	96.8	56	3.2		1,600	97.2	33	2.8	
HL	313	79.4	82	20.6		393	99.6	2	0.4	
HM	641	93.7	44	6.3		677	98.8	8	1.3	
HH	1,187	96.0	49	4.0		1,229	99.2	7	0.8	
Subjective social class					<.0001					<.0001
Low	3,278	74.6	1,144	25.4		3,569	80.2	853	19.8	
Middle	3,967	90.0	470	10.0		4,314	97.1	123	2.9	
High	2,141	92.9	175	7.1		2,299	99.1	17	0.9	
Education					<.0001					<.0001
≤Middle school	2,277	63.5	1,242	36.6		3,040	23.9	479	3.9	
High school	3,590	90.0	390	10.0		3,617	90.6	363	9.4	
≥College	3,519	95.6	157	4.4		3,525	95.1	151	4.9	
Age					<.0001					<.0001
≤29	2,653	97.3	77	2.7		2,589	94.3	141	5.7	
30–39	2,345	94.1	132	5.9		2,305	92.6	172	7.4	
40–49	1,982	89.2	234	10.8		1,975	89.3	241	10.7	
50–59	1,292	78.0	367	22.0		1,489	89.2	170	10.8	
60–69	734	60.9	464	39.1		1,061	88.6	137	11.4	
≥70	380	42.9	515	57.1		763	85.3	132	14.8	
Gender					<.0001					<.0001
Male	4,688	87.2	676	12.8		4,881	90.4	483	9.6	
Female	4,698	82.2	1,113	17.8		5,301	91.6	510	8.4	
Residential region					<.0001					<.0001
Capital	2,185	86.2	376	13.8		2,260	88.5	301	11.5	
Big city	2,800	84.7	522	15.3		2,995	90.1	327	9.9	
Small city	4,401	83.9	891	16.1		4,927	92.9	365	7.1	
Marital status					<.0001					<.0001
Single	3,458	86.1	671	13.9		3,695	89.7	434	10.3	
Married	5,928	83.7	1,118	16.3		6,487	91.9	559	8.1	
Shift work					<.0001					<.0001
Yes	381	92.5	31	7.5		380	92.1	32	7.9	
No	3328	93.7	241	6.4		3295	92.6	274	7.4	
Not employed	5677	79.8	1517	20.2		6507	90.1	687	9.9	
Smoking status					<.0001					<.0001
Smoker	2,421	89.4	287	10.6		2,423	88.9	285	11.1	
Former smoker	1,219	76.4	368	23.6		1,422	88.1	165	11.9	
Never	5,746	84.9	1,134	15.2		6,337	92.6	543	7.4	
Alcohol use					<.0001					<.0001
Yes	5,664	90.9	596	9.1		5,718	91.2	542	8.8	
Former user	791	64.3	419	35.7		1,074	86.9	136	13.1	
No	2,931	80.6	774	19.4		3,390	92.0	315	8.0	
Sensory system inability					<.0001					<.0001
Yes	51	18.3	229	81.7		218	75.6	62	24.4	
No	9,335	86.5	1,560	13.5		9,964	91.4	931	8.6	
Physical inability					<.0001					<.0001
Yes	138	14.3	807	85.7		723	75.2	222	24.8	
No	9,248	91.0	982	9.1		9,459	92.4	771	7.6	
Activity restriction 1					<.0001					<.0001
Yes	100	19.2	435	80.8		401	74.4	134	25.6	
No	9,286	88.0	1,354	12.0		9,781	91.8	859	8.2	
Activity restriction 2					<.0001					<.0001
Yes	11	4.3	255	95.7		183	67.1	83	32.9	
No	9,375	86.8	1,534	13.2		9,999	91.6	910	8.4	
Activity restriction 3					<.0001					<.0001
Yes	41	7.4	446	92.6		357	70.8	130	29.2	
No	9,345	88.2	1,343	11.8		9,825	91.9	863	8.1	
Activity restriction 4					<.0001					<.0001
Yes	183	14.8	953	85.2		882	76.4	254	23.6	
No	9,203	92.3	836	7.7		9,300	92.6	739	7.4	
Self-rated health										<.0001
Good	N/A					8,789	93.5	597	6.5	
Bad						1,393	77.0	396	23.0	
Life satisfaction					<.0001					
Good	8,789	87.1	1,393	12.9		N/A				
Bad	597	61.0	396	39.0						
Total	9,386	84.7	1,789	15.3		10,182	91.0	993	9.0	

**Fig. 1:**
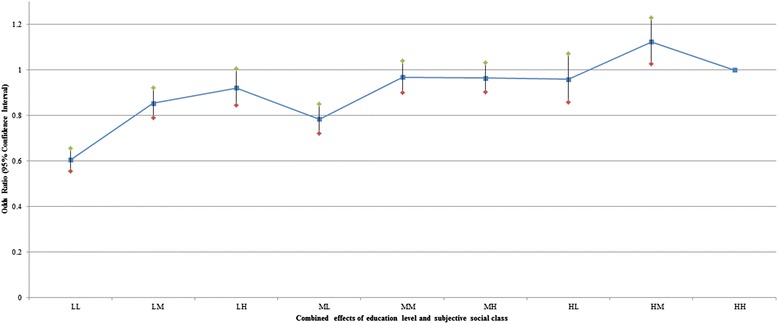
Adjusted combined effects of education and perceived social class on self-rated health. Age, gender, residential region, marital status, shift work, smoking status, alcohol use, sensory system inability, physical inability, activity restriction 1–4, life satisfaction, self-rated health, year were adjusted. Detailed results were included as Additional file 1: Table S1

**Fig. 2:**
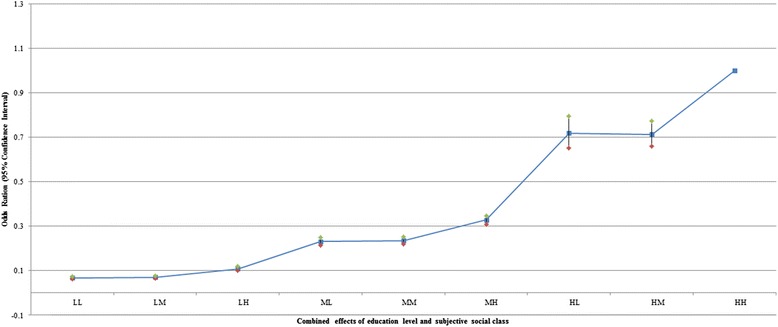
Adjusted combined effects of education and perceived social class on life satisfaction. Age, gender, residential region, marital status, shift work, smoking status, alcohol use, sensory system inability, physical inability, activity restriction 1–4, life satisfaction, self-rated health, year were adjusted. Detailed results were included as Additional file 1: Table S1

Figures 1 and 2 show the adjusted combined effects of education level and perceived social class on self-rated health and life satisfaction, respectively. Odds ratio (OR) of individuals with low education and low perceived social class was the lowest probability of self-rated health (OR: 0.604; 95 % CI: 0.555–0.656) compared to high education level and high perceived social class. The OR of individuals with low education and middle perceived social class was 0.853 times lower (95 % CI: 0.790–0.922) compared to high education level and high perceived social class. For life satisfaction, OR of individuals with low education and low perceived social class was the lowest probability of self-rated health (OR: 0.068; 95 % CI: 0.063–0.074) compared to high education and high perceived social class, and the OR of individuals with low education and middle perceived social class was 0.071 (95 % CI: 0.066–0.077). The OR of individuals with middle education and middle perceived social class was 0.235 times lower (95 % CI: 0.221–0.251) compared to high education and high perceived social class.

Additionally, we analysed the association of education level and perceived social class on self-rated health and life satisfaction, respectively (Table 3). The adjusted effect of the association of education level and perceived social class on self-rated health and life satisfaction deteriorated across the socioeconomic spectrum.

**Table 3:** Combined effects of socioeconomic stratum and perceived Social class on Self-Rated Health and Life Satisfaction

	Self-Rated Health			Life Satisfaction		
	OR	95 % CI		OR	95 % CI	
Education						
Low	0.783	0.736	0.832	0.683	0.642	0.725
Middle	1.023	0.974	1.074	0.703	0.673	0.734
High	1.000			1.000		
Perceived Social Class						
Low	0.765	0.726	0.806	0.101	0.096	0.106
Middle	0.902	0.863	0.943	0.328	0.315	0.342
High	1.000			1.000		

Adjusted for age, gender, marital status, shift work, smoking status, alcohol use, sensory system inability, physical inability, activity restriction1, activity restriction2, activity restriction3, activity restriction4, life satisfaction(self-rated health) and year

## Discussion

### Summary of main findings

In this study, our primary purpose was to investigate the combined effects of education level and perceived social class on self-rated health and life satisfaction in longitudinal models using a nationally representative sample in South Korea.

There are many research studies on the relationship between traditional socioeconomic status such as income, occupation, and education, and health outcomes. Large socioeconomic differences have been observed in self-rated health. Generally, persons of low socioeconomic status have poorer self-rated health than persons of high socioeconomic status [22, 23].

The meaning of ‘social class’ referring to SES is complex [24]. In general, indicators of SES are meant to provide information about an individual’s access to social and economic resources. Several theories have been put forth to explain observed social gradients in health [25]. Among SES, education is an important determinant of an individual’s work and economic circumstances [26], which are themselves linked to health through specific work conditions and levels of consumption. Education is also associated with health through its connection to health behaviours. The higher one’s level of education, the more likely one is to engage in a range of health-enhancing self-maintenance activities [27]. Years of completed schooling are reported with reasonable ease and reliability and are a meaningful indicator of SES for virtually all adults. Because education is typically completed early in adulthood, it serves as a marker of early life circumstances [27], and no reverse-causation problems result from linking education with health outcomes. Recently, many studies suggest that perceived social class relates to health [28] when explaining health disparities over the past decade.

However, education and perceived social class may not perfectively reflect one’s social position. Thus, a variety of research has been hampered by a lack of social class indicators. According to a study targeting a Total Hip and Knee Replacement (THR/TKR) Dutch population [29], education level (completed level of schooling) had no effect on improvement in quality of life and patient satisfaction, and had a small effect in a similar TKR population. According to a Swedish study, occupational class was also not associated with self-rated health when these other factors were accounted for [30]. Therefore, we included the gap between SES and perceived social class because it is necessary to consider the combined indicators. To the best of our knowledge, this is the first study to use self-rated health and life satisfaction to examine the combined effect of education and perceived social class of Korean adults, for which we report four key findings.

1. Individuals with a low level of education were significantly more likely to report low self-rated health, compared to individuals with a high level of education.
2. Individuals with a low education level were significantly more likely to report low life satisfaction, compared to individuals with a high education level.
3. Although in the same level of each high, middle, and low education, the possibility of an increase in self-rated health and life satisfaction increased as perceived social class increased from low to high.
4. On the contrary to this, we also saw such a trend that the possibility of an increase in self-rated health and life satisfaction increased as education level increased from low to high, although in the same level of each high, middle and low perceived social class.

Our analysis suggests that it is important to consider the impact of the combined effects on self-rated health and life satisfaction by simultaneously considering education level and perceived social class, as opposed to considering only perceived social class. In addition, our findings suggest that previous studies [31, 32] have overstated the importance of perceived social class in determining health. Our analysis using adjusted combined effects of education level and perceived social class to predict self-rated health and life satisfaction is associated more than using a single simple measure such as perceived social class to predict health outcomes.

Our results have important implications for future research and policy. As life satisfaction and self-rated health capture one aspect of wellbeing and predict future risk of objective health outcomes, further research using different indicators is needed to check the consistency of findings.

In addition, the differing magnitude of inequalities by measure of SES highlights the importance of using multiple measures when quantifying inequalities in self-rated health and life satisfaction. We recommend future research examine the impact of changes to health policy to try to unpack which policies may foster a more equitable distribution of wellbeing. Our findings suggest that mechanisms to buffer the effect of SES may help to reduce socioeconomic inequalities in life satisfaction and self-rated health.

Social relations in the form of social capital, support, and networks have been found to be important determinants of self-rated health [33, 34]. It is assumed that the quality of social interaction results in psychological reactions, which in turn affects health. In addition, life satisfaction is a multidimensional social construct that comprises more than an absence of poor health [35, 36]. The most consistent predictors of life satisfaction are health and wealth status. This finding brings together the evidence that supports the importance of health and wealth on life satisfaction [37, 38].

There are a number of strengths and limitations to this study. The strength of our study is that it is based on a very large sample that included a wide range of variables covering socioeconomic status, such as working conditions and daily life restriction. Thus, the results can be generalised to the South Korean population residing in a city region. In addition, the longitudinal design and sophisticated statistical analyses are a strength of the study. Further, we used a rather novel method to study the association of combined effects for life satisfaction and self-rated health over time.

Nevertheless, this study has some limitations that should be mentioned. First, it was based on self-report measures, which may cause systematic measurement errors (common methods variance). The longitudinal design used in this study diminishes the risk of common method bias. However, this study did not examine causality. For example, the design does not allow for identifying if one’s health effects cause one’s social economic status or vice versa [39, 40]. Second, because personality characteristics are likely to be associated with both gaps and self-rated health and life satisfaction, failure to include them in the statistical models could lead to an exaggeration of the association of interest. Third, although various studies suggest an association between social activity and health outcomes [41, 42] and life satisfaction [43], we could not control for the potential confounding of social relationships and individual partnership due to lack of information in KLIPS data. We were only able to consider physical disabilities and activity restrictions.

## Conclusions

This study shows that gap of different socioeconomic spectrum is associated with poor self-rated health and life satisfaction. Our study provides unique concrete evidence that the differing magnitude of education level and perceptions of one’s position in the social hierarchy could have important health implications when quantifying inequalities in life satisfaction and self-rated health. Therefore, policy makers need to consider how to improve the perceptions of one’s position in the social hierarchy as well as education level. In addition, researchers need to develop multi-dimensional measurement tools including education level and subjective social class. Then further research using different indicators could be used to check the consistency of findings.

## Additional file

Additional file 1: Table S1. Adjusted combined effects of Education and Subjective Social Class on Self-Rated Health and Life Satisfaction. (DOC 134 kb)
