# Supplementary material for: Combined effects of education level and perceived social class on self-rated health and life satisfaction: Results of Korean labor and income panel study wave 8-wave 15
Source: Health Qual Life Outcomes. 2015 Nov 2;13:178. doi: 10.1186/s12955-015-0375-5 (PMC4630931; doi:10.1186/s12955-015-0375-5)
Supplement: Additional file 1: Table S1. — Adjusted combined effects of Education and Subjective Social Class on Self-Rated Health and Life Satisfaction. (DOC 134 kb) [file 12955_2015_375_MOESM1_ESM.doc]

| **Additional file 1: Table S1. Adjusted combined effects of Education and Subjective Social Class on Self-Rated Health and Life Satisfaction** | | | | | | | | | |
| --- | --- | --- | --- | --- | --- | --- | --- | --- | --- |
|  | **Self-Rated Health** | | | | |  | **Life Satisfaction** | | |
|  | **OR** | | **95% CI** | | |  | **OR** | **95% CI** | |
| **Combined effects of education level and subjective social class** | | | | | |  |  |  |  |
| **LL** | | **0.604** | **0.555** | | **0.656** |  | **0.068** | **0.063** | **0.074** |
| **LM** | | **0.853** | **0.790** | | **0.922** |  | **0.071** | **0.066** | **0.077** |
| **LH** | | 0.921 | 0.844 | | 1.005 |  | **0.109** | **0.101** | **0.119** |
| **ML** | | **0.783** | **0.720** | | **0.851** |  | **0.231** | **0.214** | **0.249** |
| **MM** | | 0.968 | 0.901 | | 1.041 |  | **0.235** | **0.221** | **0.251** |
| **MH** | | 0.964 | 0.902 | | 1.031 |  | **0.328** | **0.310** | **0.348** |
| **HL** | | 0.959 | 0.859 | | 1.070 |  | **0.719** | **0.651** | **0.795** |
| **HM** | | **1.123** | **1.026** | | **1.229** |  | **0.714** | **0.660** | **0.773** |
| **HH** | | 1.000 |  | |  |  | 1.000 |  |  |
| **Age** | |  |  | |  |  |  |  |  |
| **≤ 29** | | **6.387** | **5.824** | | **7.005** |  | **0.655** | **0.599** | **0.716** |
| **30-39** | | **3.525** | **3.228** | | **3.850** |  | **0.603** | **0.552** | **0.657** |
| **40-49** | | **2.424** | **2.227** | | **2.638** |  | **0.499** | **0.459** | **0.542** |
| **50-59** | | **1.661** | **1.533** | | **1.801** |  | **0.597** | **0.550** | **0.647** |
| **60-69** | | 1.082 | 0.999 | | 1.171 |  | **0.804** | **0.742** | **0.871** |
| **≥ 70** | | 1.000 |  | |  |  | 1.000 |  |  |
| **Gender** | |  |  | |  |  |  |  |  |
| **Male** | | **1.479** | **1.400** | | **1.562** |  | **0.929** | **0.887** | **0.974** |
| **Female** | | 1.000 |  | |  |  | 1.000 |  |  |
| **Residential region** | |  |  | |  |  |  |  |  |
| **Capital** | | 1.044 | 0.994 | | 1.095 |  | **0.804** | **0.768** | **0.842** |
| **Big city** | | **1.276** | **1.216** | | **1.339** |  | **0.942** | **0.903** | **0.983** |
| **Small city** | | 1.000 |  | |  |  | 1.000 |  |  |
| **Marital status** | |  |  | |  |  |  |  |  |
| **Single** | | 1.025 | 0.974 | | 1.078 |  | **0.555** | **0.530** | **0.582** |
| **Married** | | 1.000 |  | |  |  | 1.000 |  |  |
| **Shift work** | |  |  | |  |  |  |  |  |
| **Yes** | | **1.129** | **1.027** | | **1.240** |  | **1.202** | **1.098** | **1.315** |
| **No** | | 1.010 | 0.969 | | 1.053 |  | **1.293** | **1.244** | **1.343** |
| **Not employed** | | 1.000 |  | |  |  | 1.000 |  |  |
| **Smoking status** | |  |  | |  |  |  |  |  |
| **Smoker** | | **0.850** | **0.802** | | **0.902** |  | **0.761** | **0.722** | **0.802** |
| **Former smoker** | | **0.873** | **0.813** | | **0.936** |  | **0.913** | **0.854** | **0.975** |
| **Never** | | 1.000 |  | |  |  | 1.000 |  |  |
| **Alcohol use** | |  |  | |  |  |  |  |  |
| **Yes** | | **1.195** | **1.147** | | **1.245** |  | **0.927** | **0.893** | **0.963** |
| **Former user** | | **0.685** | **0.634** | | **0.740** |  | 0.980 | 0.912 | 1.054 |
| **No** | | 1.000 |  | |  |  | 1.000 |  |  |
| **Sensory system inability** | |  |  | |  |  |  |  |  |
| **Yes** | | **0.520** | **0.437** | | **0.619** |  | 0.998 | 0.887 | 1.124 |
| **No** | | 1.000 |  | |  |  | 1.000 |  |  |
| **Physical inability** | |  |  | |  |  |  |  |  |
| **Yes** | | **0.266** | **0.241** | | **0.294** |  | 0.940 | 0.859 | 1.028 |
| **No** | | 1.000 |  | |  |  | 1.000 |  |  |
| **Activity restriction 1** | |  |  | |  |  |  |  |  |
| **Yes** | | 0.984 | 0.872 | | 1.111 |  | **0.811** | **0.737** | **0.894** |
| **No** | | 1.000 |  | |  |  | 1.000 |  |  |
| **Activity restriction 2** | |  |  | |  |  |  |  |  |
| **Yes** | | **0.711** | **0.569** | | **0.889** |  | **0.698** | **0.608** | **0.801** |
| **No** | | 1.000 |  | |  |  | 1.000 |  |  |
| **Activity restriction 3** | |  |  | |  |  |  |  |  |
| **Yes** | | **0.621** | **0.533** | | **0.723** |  | **0.838** | **0.748** | **0.939** |
| **No** | | 1.000 |  | |  |  | 1.000 |  |  |
| **Activity restriction 4** | |  |  | |  |  |  |  |  |
| **Yes** | | **0.161** | **0.148** | | **0.176** |  | **1.268** | **1.168** | **1.376** |
| **No** | | 1.000 |  | |  |  | 1.000 |  |  |
| **Life satisfaction** | |  |  | |  |  |  |  |  |
| **Good** | | **5.874** | **5.478** | | **6.298** |  | N/A | | |
| **Normal** | | **2.034** | **1.913** | | **2.163** |  |  |  |  |
| **Bad** | | 1.000 |  | |  |  |  |  |  |
| **Self-rated health** | |  |  | |  |  |  |  |  |
| **Good** | | N/A | | | |  | **3.826** | **3.587** | **4.082** |
| **Normal** | |  |  |  |  |  | **1.294** | **1.217** | **1.376** |
| **Bad** | |  |  |  |  |  | 1.000 |  |  |
| **Year** | |  |  |  | |  |  |  |  |
| **Wave 8** | | **0.764** | **0.724** | **0.806** | |  | **0.639** | **0.606** | **0.674** |
| **Wave 9** | | **0.916** | **0.869** | **0.966** | |  | **0.646** | **0.613** | **0.680** |
| **Wave 10** | | 0.985 | 0.933 | 1.039 | |  | **0.701** | **0.666** | **0.738** |
| **Wave 11** | | **1.110** | **1.053** | **1.171** | |  | **0.609** | **0.579** | **0.640** |
| **Wave 12** | | **1.091** | **1.039** | **1.145** | |  | **0.714** | **0.681** | **0.748** |
| **Wave 13** | | **0.947** | **0.905** | **0.991** | |  | **0.808** | **0.772** | **0.845** |
| **Wave 14** | | **0.915** | **0.876** | **0.955** | |  | **0.901** | **0.862** | **0.941** |
| **Wave 15** | | 1.000 |  |  | |  | 1.000 |  |  |
